# Sentiments analysis of fMRI using automatically generated stimuli labels under naturalistic paradigm

**DOI:** 10.1038/s41598-023-33734-7

**Published:** 2023-05-04

**Authors:** Rimsha Mahrukh, Sadia Shakil, Aamir Saeed Malik

**Affiliations:** 1 Institute of Space Technology, Islamabad, Pakistan; 2 Faculty of Information Technology, Brno University of Technology, Brno, Czech Republic; 3 Turner Institute for Brain and Mental Health, Monash University, Melbourne, Australia

**Keywords:** Emotion, Computer science

## Abstract

Our emotions and sentiments are influenced by naturalistic stimuli such as the movies we watch and the songs we listen to, accompanied by changes in our brain activation. Comprehension of these brain-activation dynamics can assist in identification of any associated neurological condition such as stress and depression, leading towards making informed decision about suitable stimuli. A large number of open-access functional magnetic resonance imaging (fMRI) datasets collected under naturalistic conditions can be used for classification/prediction studies. However, these datasets do not provide emotion/sentiment labels, which limits their use in supervised learning studies. Manual labeling by subjects can generate these labels, however, this method is subjective and biased. In this study, we are proposing another approach of generating automatic labels from the naturalistic stimulus itself. We are using sentiment analyzers (VADER, TextBlob, and Flair) from natural language processing to generate labels using movie subtitles. Subtitles generated labels are used as the class labels for positive, negative, and neutral sentiments for classification of brain fMRI images. Support vector machine, random forest, decision tree, and deep neural network classifiers are used. We are getting reasonably good classification accuracy (42–84%) for imbalanced data, which is increased (55–99%) for balanced data.

## Introduction

Emotions, defining our integrated feeling state due to physiological changes and sentiments, defining our positive or negative feeling underlying an opinion, play important roles in establishing successful lives both as individuals and as part of a society^1^. Our emotions and sentiments are influenced by naturalistic paradigms around us such as what we watch, listen, and read. We can choose what we watch, listen, or read, however, we are not always sure about the influence of our own choices on our emotions and sentiments. Additionally, due to influx of social media, we see, hear, and read things unintentionally, which also influence our emotions and sentiments. Social media is reported to influence mental health negatively^2,3^, especially in adolescents^4–7^. These influences on our emotions/sentiments are associated with the changes in our brain functionality. Comprehending the relationship of a stimulus, its associated emotion(s)/sentiment(s), and corresponding brain functionality may provide some biomarkers for identification and/or treatment of mental and neurological issues such as stress, depression, and ADHD.

Naturalistic stimuli such as movie, speech, and music are diverse and influence our perception, cognition, and emotions^8^. They are a normal part of our daily lives and none of us can escape the influence they have on our emotions, leading to changes in our behavior/opinion reflected in our sentiments. Naturalistic paradigm has emerged as a neuroscience approach with great potential to decipher neurodynamics in the real-world environment^9^ and can lead to unique brain findings^10,11^. Experiments involving naturalistic stimuli are better than those with task-based stimuli, which are performed in controlled environment to minimize redundant information in the stimuli^12^ and also those done in resting-state in which many subjects doze off for short intervals, introducing significant inter-subject variability^9^. Naturalistic stimuli are specifically important in affective neuroscience^13^ that deals with neural mechanism of emotions and open-access emotion datasets are created for such studies^14^.

Our emotions are complex psychological states and six emotions of happiness, sadness, anger, fear, surprise, and disgust are universally accepted^15^. Emotions and sentiments are related since sentiments are opinions of individuals, which are thought to be influenced by emotions. Sentiments can be positive, negative, or neutral and are expressed by individuals, mostly in the form of text. Sentiment analysis of various kind of texts such as tweets^16^, blogs^17^, and movie reviews^18^ is performed. Recently, studies have also started to work on sentiment analysis of movie subtitles^19^.

**Figure 1:**
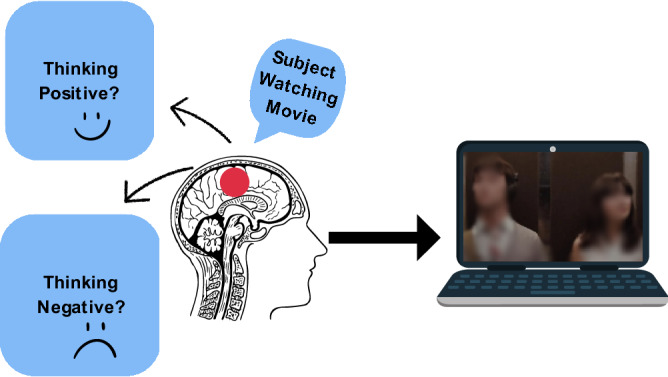
Positive and negative emotions evoked during movie watching.

Our brain functions continuously with changing activation over time and space. Changes in our brain activation can be spontaneous occurring during sleep/rest or they can be evoked by external stimuli^9^. Activation in the brain in response to a stimulus is in various regions based on the stimulus type. For example, watching a movie would activate regions in the visual cortex and the ones involved in emotion processing at the same time as shown in Fig. 1. Functional magnetic resonance imaging (fMRI) images are used to explore the functionality of our brain by detecting minimal changes in the blood flow^20,21^. It has high spatial resolution which makes it popular for the studies focusing on brain networks evolved as a result of co-activation of sub-cortical regions in the brain^22^.

Emotions are shown to be reflected in brain imaging studies using controlled models, which includes small video clips, music clips, or pictures^13^. In^23^, it is reported that naturalistic stimuli is better than ‘laboratory style’ of depending on task-based studies, which provide low test-retest reliability^24^. Naturalistic stimuli make testing and retesting more reliable, since it is more representative of the regular on-going activities of the brain.

Authors in^9^ reports that emotions are for the most part characterized as transient processes caused by inner or outside stimuli that will cause automatic changes in numerous elements of function, such as physiology, behavior, motivation, and conscious experience. Individuals experience them differently for the same stimuli and have individualized physiological responses also such as sweating, feeling nauseated etc. Consequently, the associated sentiments for individuals are also different. However, there is a portion of the emotional response due to the contents of the stimuli that is common in all subjects^13^.

This study is using movie contents (subtitles) to classify the sentiments using fMRI data to explore the extent the contents of a movie may be associated with induced emotions across all subjects. We are generating sentiments’ labels from the stimulus (movie subtitles) itself. These labels are used for sentiment classification from fMRI data to explore the relationship of stimulus, its associated sentiments, and and corresponding brain functions. To the best of our knowledge, this is the first study utilizing the subtitles-generated labels for fMRI images’ classification. In this study, we are using most popular lexicon-based sentiment (VADER, TextBlob) and neural network (Flair) sentiment analyzers. Using the labels generated with these analyzers, we classify the fMRI data with support vector machine, random forest, decision tree, and deep neural network classifiers .

Specific contributions of our study to the field are:

1. Automatic generation of sentiment-based labels from sentiment analysis of movie subtitles.
2. Sentiment classification from fMRI data using labels generated from movie subtitles.

The paper is organized as follows: “Introduction” Section contains Introduction and Literature Review. “Methodology” Section explains Methodology. “Results” Section contains Results followed by Discussion in “Discussion” Section. “Conclusion” Section concludes the paper.

### Literature review

Emotion/sentiment classification using neuroimaging data such as electroencephalogram (EEG), fMRI, and their combination collected during movie watching or music listening is performed in many studies^25^. For example,^26^ used movie clips to evoke emotions and classified them using Support Vector Machine (SVM) on 128 channel EEG data from six subjects. The study reported that subject-independent features can be used to successfully classify emotions from EEG for brain-computer interface. In another study^27^, using Chinese movie clips targeted to illicit specific emotions, the authors constructed EEG-based emotion recognition models for positive, negative, and neutral emotions using deep belief networks (DBNs).

Many studies used two popular open−access EEG datasets; DEAP (Dataset for Emotional Analysis using Physiological Signals) and SEED (SJTU Emotional EEG Dataset) for emotion/sentiment analysis. DEAP contains EEG data from 32 participants collected while each of them watched one-minute long music videos^28^. For SEED^29^, twenty participants assessed their emotions while watching video clips by score (1–5) and by keywords (positive, negative, and neutral). In^30^ long-short term memory (LSTM) consisting of two LSTM layers, dropout layer, and dense layer was applied on DEAP dataset for emotion classification. An average accuracy of 0.865 was obtained. Another study^31^ provided comparative analysis of machine- and deep- learning techniques for binary classification of six basic emotions (anger, disgust, fear, joy, sadness, and surprise) using DEAP dataset.

In^32^, EEG-based emotion recognition system was developed using the time dependency property of emotion recognition from SEED dataset using ensemble learning. Wang et al. proposed^33^ electro-frequency distribution maps with short-time Fourier transform along with Convolutional Neural Network (CNN). Using deep transfer learning, they received a training accuracy of 90.59% on SEED testing accuracy of 82.84% DEAP.^34^ developed an easy to use device for brainwave readings through the use of long short-term memory (LSTM) networks to recognize sentiments as either positive, negative, or neutral is now available. Models were evaluated on both DEAP and SEED datasets.

In addition to EEG, fMRI is also used for analysis of human emotions.^35^ studied continuous changes of affective valence during movie watching. Movie valence scores were obtained from one group and valence rating from fMRI of another group was predicted using these scores with Pearson correlation. Twelve ten-minutes emotion-evoking movie clips were shown to 52 individuals to explore their functional connectivity (FC) profiles in^36^. Predictive FC profiles for sustained sadness and happiness prediction were developed using SVM and subjective feedback. Last 200 s of the movies were found to have stronger predictive power compared to early stimulation. In^37^, low-level audiovisual and high level fMRI FC features were combined using mulitmodal Boltzmann machine (DBM) for classification using SVM. It was reported that joint representation outperformed low-level audiovisual features. In^38^, Hidden Markov Model (HMM) was to investigate the changes in brain activity while individuals watched long-movie clips. This helped them analyze how emotions are reflected in the brain dynamics. Inter-subject neural activity was aligned using dynamic hyperalignment algorithm (dHA), which resulted in more consistent temporal HMM states across subjects. Data from StudyForest project consisting of 15 participants watching 2 hours of the movie was used. Different set of 12 healthy volunteers provided emotion ratings of the six basic emotions. Using specificity and sensitivity of HMM common response across subjects and time-varying brain states during movie watching were identified.

Emotion classification for mental disorders is also performed using brain activation data. In^39^, authors reported use of SVM and ANN for successful classification of patients with autism spectrum disorder (ASD) and healthy brain patterns with EEG data.^40^ used functional connectivity on EEG frequency bands to differentiate between ASD patients and healthy controls.^41^ used SVM classifier with linear kernel to identify brain structural features along with functional features to identify social anxiety disorder (SAD) using both resting-state and video fMRI data. Static and dynamic inter-subject correlation was used in^42^ to examine the association of brain function in depressive symptoms of children and adolescents with emotional movie fMRI data.

In studies to analyse emotions from brain activation data collected during naturalistic paradigm, labels for the emotional contents in the stimuli are subjective since they are generated by humans. Furthermore, these labels contain information about the overall emotional contents of the stimuli and not about their variability over time. Consequently, time-varying influence of these stimuli cannot be studied. One way to reduce the subjective influence is to generate emotional labels of the stimuli by analyzing the contents of the stimuli. For example,^43^ used connotative features of movies along with each user’s previous responses to similar stimuli for their emotion prediction. In^44^, emotions are extracted from videos in two steps. In the first step, CNN was trained to classify static images containing emotions and in the second step Recurrent Neural Network (RNN) was trained on higher features to predict a single emotion from the entire video. Another study^45^ used mutilmodal deep learning approach using visual information and spatio-temporal aspects of the videos to assign one single emotion to the whole video. These approaches are effective in identifying the overall emotion in a video, however, they still don’t provide the information about the variability in the emotional contents of a video over time.

Sentiment analysis uses natural language processing (NLP) and text analysis techniques to extract subjective information from the text^46^. A basic task in sentiment analysis is classifying the polarity of a given text (positive, negative, or neutral) and advanced task can be to identify emotions in the text^47^. Sentiment analysis is an approach of natural language processing (NLP) to identify emotional tone from a body of text. Strategies for sentiment analysis (SA) can be classified as AI-based, Lexicon (vocabulary)-based, and hybrid. Significant work has been done for SA of printed information by using any of these techniques. Cambria in^48^ recommended that the primary errand of SA is acknowledgment of emotion and polarization detection. This study distinguishes three wide kinds of SA approaches: (1) knowledge based procedures in which the text is classified into affect categories based on presence of unambiguous affect words such as happy, sad etc. (2) Statistical based that deals with annotated data and use SVM and deep learning for classification of text. (3) Hybrid methodology that utilizes both vocabulary and machine learning together.^49^ uses mining sentiment to discover sentiment of political opinion tweets in 17 provinces which held US presidential elections in 2018. In^50^, the results of sensory exploration are analyzed in more detail using correlation analysis and sentiment analysis. The method used here for correlation analysis is Pearson correlation, while sentiment analysis used Naive Bayes and the C5.0 methods. Performance of Naive Bayes^51,52^ and C5.0 methods are evaluated based on accuracy, precision, and recall. C5.0 is an interactive calculation that allots diverse weights to training and testing information in each iteration^53^.

**Figure 2:**
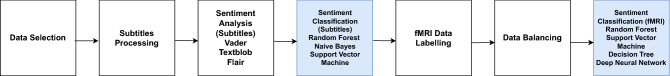
Block diagram of the study sequence.

## Methodology

We hypothesize that the emotional aspect of movies change over time influencing the emotional and associated brain states of individuals dynamically. Furthermore, since a movie comprised of many components such as audio, video, and dialogues (subtitles), all of these should influence the emotional state of an individual in addition to his/her own pre-stimulus emotional state.

In order to perform sentiment classification of fMRI data collected during movie watching, we decided to use open-access data. However, none of these dataset provided time-based sentiment labels along with the fMRI data so we decided to generate these labels over time (dynamically) using the features of the movie itself. For this purpose, we used movie subtitles provided along with the movie. We classified sentiments using fMRI data with these labels to test our hypothesis. Our target was to perform classification with two (positive, negative) and three (positive, negative, neutral) sentiments using both lexicon-based (rule-based) and machine learning sentiment analyzers. Figure 2 shows the block diagram of our study. Description of various blocks is provided below.

### Data selection

A publicly available dataset (Naturalistic neuroimaging database (NNDb)^23^) was used in which 86 participants went through behavioral testing and watched one of 10 movies (of different genres) while fMRI was acquired. The time series provided are of very high quality with a good SNR. It was taken in a well-organized place and also with low movement of the participants. For functional and anatomical images, a 1.5 T Siemens MAGNETOM Avanto machine with a 32 channel head coil (Siemens Healthcare, Erlangen, Germany) was utilized. They have employed the use of multib and EPI with a TR of 1 s and TE of 54.8 ms, a flip angle of 75$^\circ$ and 40 interleaved slices with an isotropic resolution of 3.2 mm. Additionally, the ’leak block’ option was enabled to prevent cross-slice aliasing which can occur in multi-band processing with a 4x factor and underlying in-plane acceleration. For full brain coverage, the slices were angled manually. The EPI scanning had a software limitation of a continuous one hour, thus each movie had to be interrupted at least once. The amount of volumes collected from each participant varied significantly, ranging from 5470 to 8882, depending on the movie they watched. Movie annotations were provided and we used word annotation file to extract our subtitles data, which was further used to label our fMRI timeseries.

This dataset was chosen for this study due to a number of reasons. First of all, NNDb is relatively new and unexplored open-access database of 86 participants watching 10 full length movies of different genres evoking different types of emotions/sentiments. In addition to providing high quality neuroimaging data (fMRI and MRI), NNDb also provided the list of all words in the movie. It is hypothesized that the sentiments of the person watching the movie may be influenced by subtitles, given that they are composed of words carrying semantic information. NNDb data is fully preprocessed, which provides standardization and make the results reproducible across studies. In case of raw data, preprocessing steps and chosen toolboxes/software influence the final results making it difficult to compare the results across studies even for the same data. Preprocessing pipeline of AFNI’s afni_proc.py was implemented [https://openneuro.org/datasets/ds002837/versions/2.0.0]. Anatomical MRI preprocessing included skull stripping, MNI alignment, WM/CSF mask extraction, and segmentation. For functional MRI the preprocessing involved time shifting, despiking, MNI alignment, volume registration, smoothing, and artifacts removal using ICA.

This is a pilot study to explore the possibility of using subtitles’ extracted labels for classification of the sentiments from fMRI data. In order to keep the study relatively simple, we decided to use data from just one movie. However, in order to have large number of subjects, we selected a movie that was watched by largest number (20) of participants in the database. Another reason for selecting this movie was the fact that its contents are diverse and the subtitles provided are not just from the dialogues but from the background narration/commentary, too. We rationalize that if our hypothesis is true for sentiment classification from such diverse contents, it will be true for movies having more synchronized contents containing only dialogues without narration/commentary. we are using the first 30 min of the movie data and our rationale for using only first 30 min was the fact that movies do not induce very strong emotions/sentiments at the start and getting high sentiment classification accuracy for first 30 min would suggest having better results for the whole movie. In future, we plan to add more movies data in the analysis and use full movie data.

### Subtitles processing

Movies dialogues (subtitles) were provided in a word annotation file by NNDb from where we extracted the sentences. In annotation file (.csv file), each dialogue was given word by word along with each word’s onset and offset timings based on the dialogue delivery in the movie. It is important to note that the subtitles did not match perfectly with the audio track. NNDb annotated the words automatically and matching level of the subtitles is also provided [https://www.naturalistic-neuroimagingdatabase.org/annotations.html]. In order to perform sentiment analysis, the conversion of words was done into coherent sentences. For this purpose, we watched the movie with subtitles, constructed sentences for every dialogue, and placed ‘a full stop’ at the end of each sentence indicating ending of a dialogue. Duration of a dialogue (sentence) was computed by using the onset and offset timings of each word in that dialogue. Total number of words and time along with the statistics (smallest, longest, mean, and standard deviation) for the subtitles are provided in Table 1.

**Table 1:** Subtitles statistics for length & time duration of the sentences.

	Smallest duration	Longest duration	Mean length	Standard deviation
Words (2768)	1 word	37 words	6.05 words	4.81 words
Time (1744)	1 s	67 s	3.8 s	5.5 s

**Figure 3:**
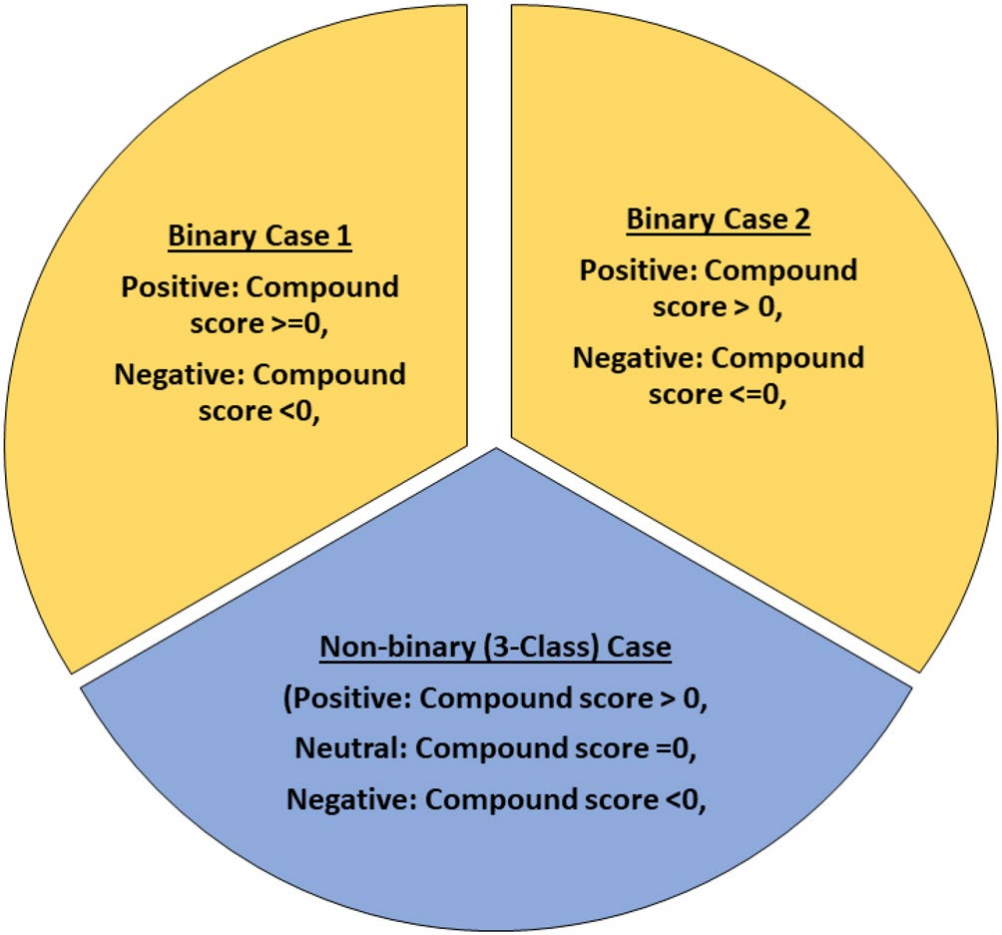
Three cases of sentiment polarities distribution (classes). Binary case 1: positive polarity (class) contains both positive & neutral sentiments. Binary case 2: negative polarity (class) contains both negative & neutral sentiments. Non-binary (3-Class) Case: positive, neutral, and negative polarities are treated as separate classes.

### Sentiment analysis

After formation of the sentences, the next step was to perform sentiment analysis on these sentences. For this purpose, both lexicon- and machine-learning- based sentiment analyzers from the domain of natural language processing (NLP) were used. Lexical approach is a simple and most commonly used approach to analyze sentiments from textual data^54^. In this approach, a sentiment lexicon (dictionary) is built from the lexical features (words), which are labeled based on their context free semantic orientation. This approach does not need any training or machine learning models but it needs the words to be in the dictionary for processing. However, machine-learning based analyzers train a model that can be used on new words, too.

We used three sentiment analyzers used from natural language processing, namely; (1) VADER (Valence Aware Dictionary for sEntiment Reasoning), (2) Textblob, and 3) Flair. VADER and Textblob are lexicon-based sentiment analyzers and compute polarity of a sentence based on the weights of its words from the lexicon^55,56^. However, they differ since in addition to lexicon, VADER also takes into account the emotional intensity of the text based on heuristics such as punctuation, emojis, and capitalization. The score of a text in VADER can be computed by adding up the intensity of each word within the text^57^.

VADER depends on a word reference that works by using dictionary that would further do the mapping of lexical features to the intensity of emotion, known as sentiment scores^58^. VADER assigns each word with either positive or negative polarity based on its own built-in dictionary, which contains sentiments associated with various words. Afterwards, it computes an overall score based on these values by taking into consideration aspects such as intensity and context within the sentence. The intensity of a word, also known as its sentiment strength, is the degree to which it expresses emotion or opinion. Polarity refers to whether the sentiment expressed in a word is positive or negative. VADER is an algorithm that measures both intensity and polarity of words based on their context within a sentence. VADER is well trained to understand the setting words for example if the text is ‘did not love’ which is actually giving a negative sentiment, VADER understands it and associate a negative articulation with it. It also stresses on the accentuation of capitalization and punctuation, such as “ENJOY”.

TextBlob is a rule-based sentiment analyzer that uses a set of pre-trained classifiers. Rule-based sentiment analysis is a type of sentiment analysis that uses predefined rules and dictionaries to classify the sentiment of text. The rules can be based on keywords, phrases, or other linguistic features. It gives justifiable results with respect to natural language processing assignments that incorporates sentiment analysis, classification, interpretation and more^59,60^. It is commonly used, which classifies whether a sentence or word is positive or negative. TextBlob’s sentiment analysis feature uses a movie reviews corpus to classify phrases by polarity (positive or negative). Sentiment analysis includes deciding a writer’s state of intellect or feelings. For example, if you analyze the sentence “I love going to the gym” using TextBlob, it would assign a positive polarity score to each of the words in the sentence and then calculate an overall sentiment score for that sentence. The result would be a positive sentiment with a higher than average confidence level.

Flair uses a machine learning approach and is based on a character-level LSTM neural network that takes sequences of words and letters in consideration when predicting sentiments. Flair has an easy mechanism which uses vector representations of words to ascertain sentiment annotations based on the sequence of those words^61^. It contains a capable library that permits clients to utilize and combine different word and document embedding. Based on the corpus, it analyzes and describes the speaker’s demeanor. Compared to other NLP bundles, Flair’s emotion classifier is based on a character-level LSTM neural arrange that takes into consideration character and word sequences when making forecasts. Flair has pre-trained models through which it performs sentiment analysis. In this study, we applied two such models (https://github.com/flairNLP/flair), and termed them as English-Sentiment (E-Sentiment) and Sentiment-Fast (F- Sentiment). Both differ in terms of their architecture only, E-Sentiment is based on LSTM while F- Sentiment is trained on RNN model^62^. They are both trained on large corpus of English text. The main reason for selecting these two different implementations of Flair was to explore if the underlying model would have much difference in corresponding classification results.

#### Sentiment score calculation

Python’s NLP toolkit (NLTK) was used for sentiment analysis. We provided the sentences constructed from the subtitles as input to the toolkit. Being lexicon-based, both VADER and Textblob followed the same steps for analysis. The first step was sentence tokenization that is the process of breaking a sentence into smaller pieces called ’tokens’. For example, the sentence ’This is an interesting study’, would be broken in five tokens; ’This’, ’is’, ’an’, ’interesting’, ’study’. Tokenization was followed by compound scores computation in which each sentence’s score was computed based on the sentiment weights of its constituent words. Next step was computation of compound score, which is normalized sum of positive, negative, and neutral scores of all words. The normalized sum lies between $-$ 1 and + 1, in which scores close to + 1 indicate positive sentiments and vice versa. Based on compound scores studies decide about the polarity of the sentences. Some studies consider three polarities in which positive compound score is taken as representative of positive sentiments, negative compound score is taken as representative of negative sentiments, and a compound score of zero is taken as neutral. Some studies define just two polarities (positive and negative) and merge compound score of zero in one of them. In this study there are three different scenarios explored for polarities as shown in Fig. 3. For Binary Case 1, we are considering neutral sentiments (polarity = 0) as positive, while for Binary Case 2, we are considering them to be negative.

In case of Flair, the model used was trained on movies reviews (IMDB) database and performed binary classification only by assigning positive or negative polarities to each sentence.

#### Similarity check

All the three sentiment analyzers used in our study have their own special purposes; VADER works perfectly for social media contents, for example, Twitter based tweets. However, TextBlob works best for formal language, while Flair is trained on IMDB data and offers pre-trained models. None of them is specifically designed for sentiment analysis of movie subtitles and no subtitles specific sentiment analyzer is available. Consequently, we needed to do performance check of the three chosen sentiment analyzers. For this purpose, a simple algorithm was developed for similarity check of their results, which is described below.

Let the polarities from two sentiment analyzers *SA*1 and *SA*2 are given by two vectors $V_{SA1}$= $\bigl \{V_{SA1i}\bigr \}_{i=1}^n$ and $\bigl \{V_{SA2i}\bigr \}_{i=1}^n$. We computed the similarities $(S_{12})$ between these two vectors as given in Eq. (1):

1 $$\begin{aligned} \begin{aligned} S_{12}&= \sum _{i=1}^{n} Sim(V_{SA1}, V_{SA2}), \text {where }\\ Sim(V_{SA1}, V_{SA2})&= {\left\{ \begin{array}{ll} 1, \text {for } V_{SA1i}=V_{SA2i}, \textit{i} \text{= } \text{1, } \text{2,...n } \\ 0, \text {Otherwise } \end{array}\right. } \end{aligned} \end{aligned}$$

Positive polarities were assigned value of 1, neutral were assigned a value of zero, and negative were assigned a value of -1 in these vectors. Equation (1) provided the total similarity scores between any two polarity vectors. This process was repeated pairwise for each pair of analyzers. The pair having the largest sum was the one having the largest similarity score. All of this processing was done using MS EXCEL.

### Sentiment classification (subtitles)

After successful generation of sentiment based polarities from all the three sentiment analyzers, we classified the subtitles by assigning sentiment labels based on these polarities. The reason was to validate the use of polarities as labels for sentiment classification from subtitles before using them as labels for classification using fMRI data. For classification, the data was either divided in two classes (binary cases) or in three classes (3-Class) case. Sentiment labels (neutral = 0; negative = 1, positive = 2) were assigned to each sentence. Two basic classifiers were chosen, namely; (1) Random forest (RF) and (2) Support vector machine (SVM) for subtitles classification. RF is an ensemble learning method which works by producing number of decision trees during the training time and its accuracy does not get affected by over-fitting. SVM is a linear classifier that work on the basis of margin maximization and very effective in high dimensional spaces. These two classifiers were selected since they have been used in many studies for sentiment classification after labeling with VADER and/or Textblob^63–69^. The data was split into train-test split where training was done on 70%, testing on 20%, and validation on 10% of the subtitles.

### fMRI data preparation

Next, fMRI data was prepared for classification. For this purpose, fMRI data features were needed along with their labeling. Both of these steps are explained below.

#### fMRI features

We used fMRI time series from various sentiments (emotions) related regions-of-interests as features for the classifiers. For our study, three happiness regions as positive, which were left anterior cingulate cortex (L_ACC), right superior temporal gyrus (R_STG), and left cerebellum (L_cere) were considered. For negative emotion data also three regions were used, which were left medial frontal gyrus (L_medFG), right inferior frontal gyrus (R_IFG) and left caudate head (L_ch). These regions were identified based on an earlier study^70^. This study identified these regions based on an earlier meta-analysis of task-induced emotion activation^71^. Seeds (in MNI coordinates) for the regions-of-interest (ROIs) were taken from^70^ and we defined spherical regions of 5mm radius around the seeds as our regions-of-interests (ROIs). Time series of all the voxels in an ROI was extracted using AFNI software.

#### fMRI data labeling

In order to utilize sentiment analyzers generated labels on fMRI data, we needed to assign sentiment labels to each fMRI image volume, which were recorded at every 1 s. Labels were assigned to each fMRI volume based on the length of the sentences. In order to do that, the time duration of each sentence was recorded and duration of fMRI data corresponding to each sentence was assigned the same sentiment label as the sentence. For example, suppose the sentence was ‘story of boy meets girl’ and its duration was 8 s and its generated label was positive. In order to assign sentiment labels to fMRI data, 8 s of fMRI data (8 volumes since fMRI TR = 1 s) corresponding to this specific sentence was taken and assigned positive label. If there was a sentence with a duration value in decimal, that value was applied with the function ‘ceil()’, which would cover the whole of 1 s with the same label. For example, if a sentence was 8.3 s long, taking time of 8 full fMRI volumes and 0.3 of the ninth volume, too. This issue was handled by applying ceil() function that would assign the label of this sentence to nine fMRI volumes instead of eight. Similarly, some sentences (dialogues) had gaps (silence), for example, if one dialogue ended at 61–64 s and next started from 66 s then $66-64$ = 2 s was a gap. Previous data label was assigned to fMRI volumes during such gaps. These steps generated some errors but they were unavoidable for data and labels alignment.

#### fMRI features matrix

**Figure 4:**
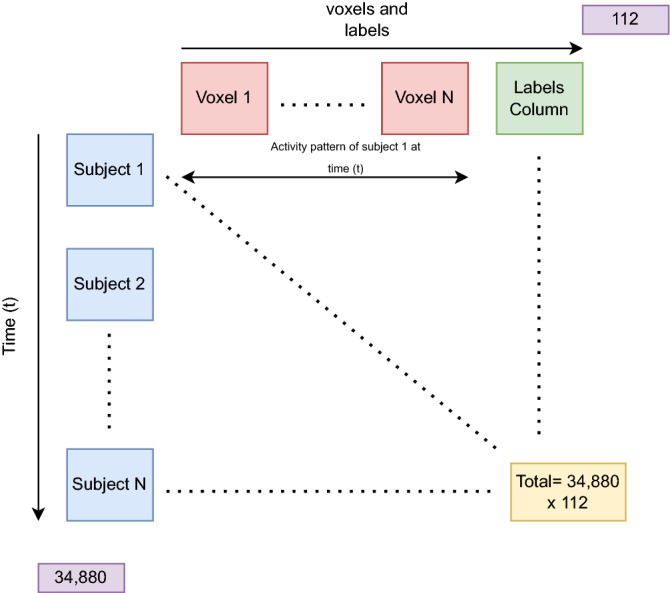
Data matrix for input to the classifiers. Brain activity pattern at each time point (rows in the matrix) is an input feature vector. Last column contains subtitles-generated labels.

For current study, we extracted approximately first 30 min (1805 fMRI volumes) of each subject’s movie fMRI data for further processing. However, the subtitles are provided after 61 s from start of the movie so we discarded first 61 s, leaving 1744 s of data from each subject. These 1744 s of fMRI volumes were labeled based on the sentence polarities from different sentiment analyzers as discussed in “fMRI data preparation” Section. Total number of voxels for each subject were 111 comprising 19 voxels from L_ACC, 20 from R_STG, 20 from L_cere, 20 from L_medFG, 16 from R_IFG, and 16 from L_ch. The final features matrix for input to the classifier had 34,880 rows (1744 * 20) and 112 columns (111 voxels + 1 labels) as shown in Fig. 4. We are taking fMRI activation of all ROIs in the sentiment network (containing 111 voxels from sentiment related regions of the brain) at a time point as the feature vector at that time point. These activations are forming the activity patterns in response to the sentiments evoked by the movie scenes. For example, if at a time point t, the sentiment is positive them all of these voxels would be activated to form activation pattern associated with positive sentiments. Consequently, combined activation patterns (in space) over all the time points are feature vectors and each of these feature vectors are labeled using the NLP-generated labels.

### fMRI data balancing

As mentioned in “Sentiment analysis” Section, Both binary and 3-Class labels were generated. (see Fig. 3). Consequently, There was imbalanced data for some of the cases such as for Binary Case 1 (neutral labels were taken as positive), the number of negatively (minority class) labeled sentences were a lot less than the positively (majority class) labeled ones for both VADER and TextBlob. The number of positive, negative, and neutral instances/labels for imbalanced data is shown in Table 4. One of the major issues in highly imbalanced data (for example 90% majority class and 10% minority class) is that the classifier will be accurate 90% of the times by classifying each input as belonging to the majority class. Initially, we did the classification with imbalanced data but did not get high classification accuracy. Consequently, the data was balanced by over-sampling (increasing the minority class data) by using two basic methods.

Two over-sampling methods applied were; (1) Random over-sampling (ROS) and (2) Synthetic Minority Over-sampling Technique (SMOTE)^72^. ROS is very simple over-sampling technique in which the samples from the minority class are replicated to increase the size of minority class. However, SMOTE brings variety to data by producing “synthetic” illustrations instead of over-sampling with substitution. For SMOTE, To over-sample the minority class, synthetic examples are generated along the lines connecting any or all of the closest neighbours from that particular group. This helps to equalise data and to make sure minority classes are adequately represented in a dataset^73^. All the labels were balanced by applying oversampling methods in python using imblearn() function. Results for both imbalanced and balanced data are reported in this study.

### fMRI classification

In order to compare the results of fMRI classification, we used two of the same classifiers as were used for subtitles classification (RF and SVM). We also used two additional classifiers, which are decision tree (DT) and deep neural network (DNN) classifiers. DT classifier are known for capturing illustrative decision-making information when they are provided with data. We customized the DNN by choosing best activation function, optimizer, and loss function for optimal performance. A study^74^ showed that SVMs are very reliable and accurate approach that is adaptable in their use. SVM differs from other classifiers in a way that it can be learned and used for training thousands of characteristics in a reasonable amount of time^75^. All of these classifiers are used in emotion/sentiment studies as explained in “Literature review” Section. Default parameter values were used for all classifiers from Python except DNN.

#### Deep neural network

Our deep neural network had one input layer followed by 7 hidden layers [100, 90, 70, 50, 30, 20, 10] and one output layer. For Binary cases, sigmoid activation function was used and for 3-Class case, Softmax function was used at output layer, respectively. ReLU function was used with each hidden layers. For loss calculation, we have used binary cross entropy and sparse categorical cross entropy for binary and 3-Case situations, respectively. We have used Adam as an optimizer, which is the most efficient one and trains a network in less time. We used binary cross entropy for 2 classes (Binary Case 1 and Binary Case 2), while sparse categorical cross entropy is used for 3-Class Case classification. The loss computed at every iteration decreased till the last epoch bringing it close to zero.

## Results

In this section, we present results of our analyses that included binary as well as 3-Class labels for sentiment classification. For binary case in all three sentiment analyzers of VADER, TextBlob, and Flair, only positive and negative labels are considered. For 3-Class case, we have three sentiments labels (neutral, positive, and negative) extracted by using VADER and TextBlob only because Flair performs binary analysis only. Classification with subtitles was performed first (see “Sentiment classification (subtitles)” Section for details). Later on, We used these labels for sentiment classification from fMRI data and the classification results are reported in terms of classification accuracy, precision, recall and F1 scores. For imbalanced data, the accuracy was too poor with a range for 50–70 percent in binary case and 30–40 percent in 3-Class case. Results of subtitles classification and fMRI classification are presented in this section.

### Similarity scores

**Figure 5:**
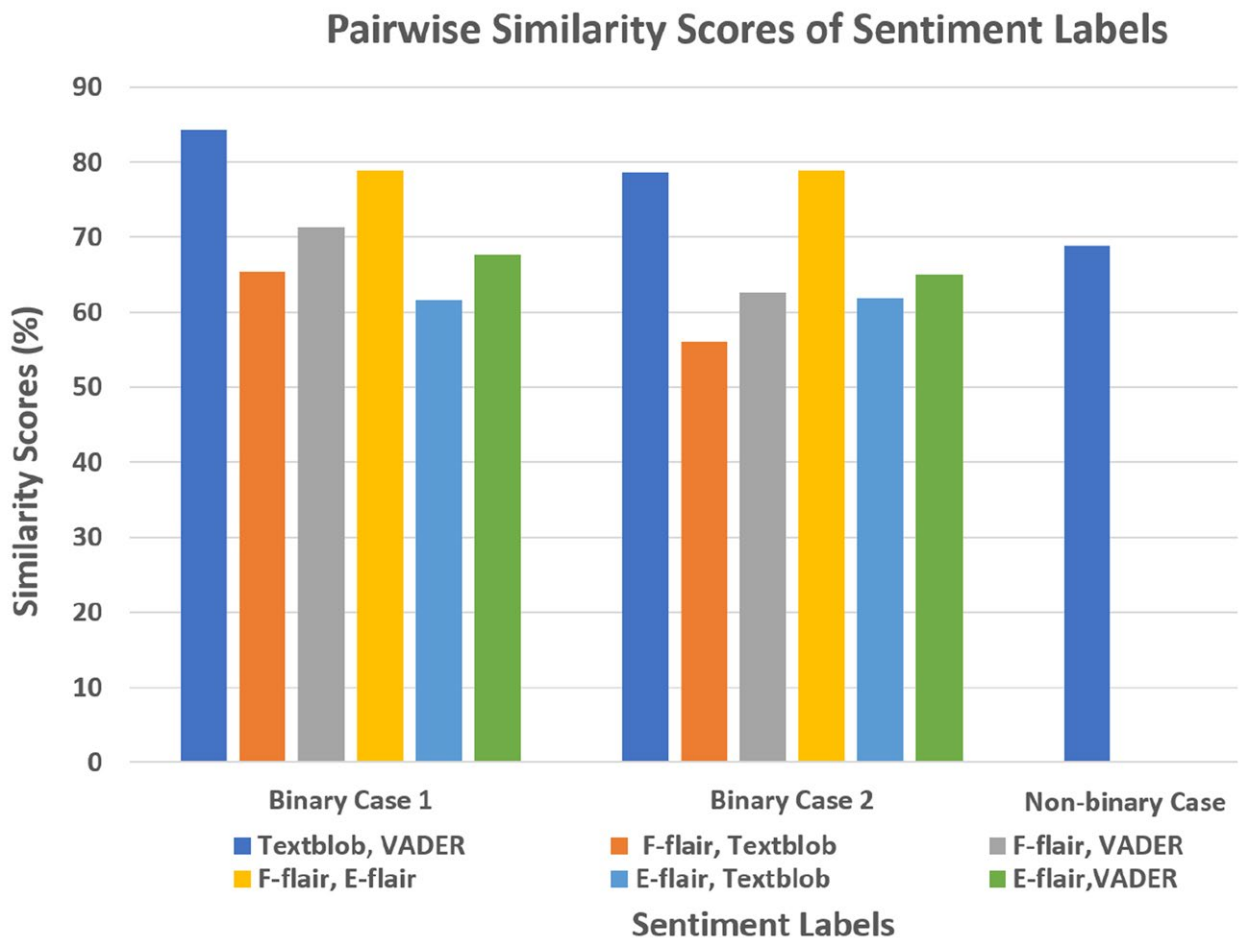
Pairwise similarity scores of the labels generated from movie subtitles.

Figure 5 shows pairwise similarity scores of the labels generated using different sentiment analyzers. In the figure, we have two sets of six results (one set for Binary Case 1 and other for Binary Case 2) for pairwise comparison of the four algorithms. It should be noted that all pairwise similarities are above 55% and the best similarity was between TextBlob and VADER for both sets followed by the similarity between E-Flair and F-Flair. Since Flair does not produce neutral labels so for 3-Class Case (positive, negative, neutral), similarity scores were computed for VADER and TextBlob only. For 3-Class Case, the polarity of zero is taken as neutral sentiment and the similarity score between Textblob and VADER is almost 70%.

### Subtitles classification

In order to validate the labels generated with sentiment analysis of movie subtitles, we classified the subtitles using these labels. RF and SVM classifiers are used and the accuracy results are shown in Table 2. Overall the classification accuracy is higher than 70% for binary cases for both classifiers. It can be observed that RF is providing better classification accuracy (76–89%), while for SVM the classification accuracy range is 68–88%. Furthermore, the classification accuracy for two sentiments (binary cases) is higher compared to three sentiments (3-Class) regardless of the classifier used. Additionally, for two sentiments case also, the accuracy was more for Binary Case 1 compared to Binary Case 2. The only exception to this rule is VADER in RF classifier. E-Flair and F-Flair, classifying on two sentiments, performed similarly for both classifiers and had accuracy relatively less than the other methods. It should be noted that this high classification accuracy results are obtained for highly imbalanced labels as shown in Table 3 and data balancing is expected to improve the classification accuracy.

In order to check if the results are indeed due to the correct labeling of the data from the subtitles, we performed the classification using shuffled labels, which are expected to mis-predict/mis-classify sentiments from both subtitles and fMRI. We performed randomization using a built in function of Python to our column of labels and applied them to classify the sentiments using both subtitles and fMRI. The results for subtitles classification with randomized labels are shown in Table 4.

**Table 2:** Accuracy for subtitles classification using subtitles-generated labels.

	Binary class 1		Binary class 2		Flair		3-class case	
	VADER	Textblob	VADER	Textblob	F-sentiment	E-sentiment	VADER	Textblob
Random forest	**0.87**	**0.89**	**0.85**	**0.85**	0.76	0.76	0.76	**0**.**8**
SVM	**0.88**	**0.87**	**0.85**	0.75	0.73	0.73	0.73	0.65

The values with accuracy are given in bold.

**Table 3:** Count of imbalanced class labels for all sentiment analyzers.

	Binary class 1		Binary class 2		Flair		3-class case	
	VADER	Textblob	VADER	Textblob	F-sentiment	E-sentiment	VADER	Textblob
Positive	27080	29260	15560	14000	22300	19920	15560	14000
Negative	7800	5620	19320	20880	12580	14960	7800	5620
Neutral	0	0	0	0	0	0	11520	15260

**Table 4:** Accuracy for subtitles classification using subtitles-generated shuffled labels.

	Binary class 1		Binary class 2		Flair		3-class case	
	VADER	Textblob	VADER	Textblob	F-sentiment	E-sentiment	VADER	Textblob
Random forest	0.52	0.52	0.42	0.42	0.42	0.52	0.41	0.42
SVM	0.55	0.55	0.48	0.48	0.55	0.48	0.29	0.29

### Imbalanced data classification

Data imbalance is identified based on unequal number of data points in different classes^76^. In our study, the imbalance was due to the different number of negative, positive, and neutral time points based on the labels extracted from the subtitles. Table 5 shows the classification results of three classifiers using imbalanced fMRI data. RF and SVM classifiers were used for comparison with subtitles classification results. We added decision tree (DT) and deep neural network (DNN) classifiers to get more diversified results. However, for DNN classification accuracy was very low due to which the results are not added in the table. It can be observed that RF and SVM are giving almost the same accuracy range (43–84%) and performing better than DT (33–77%). Similar to the subtitles , 3-Class classification performed worse than the binary classification. Furthermore, in this case also the best performance was for Binary Class 1. Contrary to subtitles classification, Binary Class 2 performance was not that good in this case. TextBlob performed best for RF and SVM, while VADER outperformed it for DT. Contrary to the subtitles’ classification, E-Flair and F-Flair performed differently for fMRI classification with F-Flair doing better than E-Flair.

**Table 5:** Accuracy for fMRI data classification with imbalanced class labels.

	Binary class 1		Binary class 2		Flair		3-Class	
	VADER	Textblob	VADER	Textblob	F-sentiment	E-sentiment	VADER	Textblob
Random forest	0.77	**0.84**	0.53	0.58	0.63	0.56	0.43	0.42
SVM	0.77	**0.84**	0.56	0.6	0.64	0.57	0.44	0.43
Decision tree classifier	0.63	0.71	0.50	0.52	0.36	0.38	0.54	0.51

The values with accuracy are given in bold.

### Balanced data (random oversampling) classification

Table 6 shows the results of fMRI data classification for data balanced with random oversampling (ROS). The results are reported for both train-test split and for k-cross (k=10) validation. In can be observed that the accuracy difference between train-test split and k-cross validation is quite small (± 0.01) for all results. It can also be observed that Binary Case 1 performed best for all classifiers for both VADER and TextBlob generated labels. TextBlob performed better than VADER in this case having best accuracy (99%) for RT. For rest of the results also, TextBlob either performed as good as VADER or better than it. F-Flair performed better than E-Flair for all the classifiers.

**Table 6:** Accuracy for fMRI data classification with balanced (Random oversampling) class labels.

		Binary class 1		Binary class 2		Flair		3-Class Case	
		VADER	Textblob	VADER	Textblob	F-sentiment	E-sentiment	VADER	Textblob
Random forest	Train-test split	**0.95**	**0.99**	0.59	0.68	0.77	0.64	0.63	0.64
	k-fold validation	**0.95**	**0.89**	0.59	0.67	0.76	0.6	0.63	0.64
SVM	Train-test split	0.77	**0.81**	0.56	0.59	0.62	0.56	0.52	0.5
	k-fold validation	0.77	**0.81**	0.56	0.6	0.65	0.58	0.52	0.52
Decision tree	Train-test split	**0.82**	**0.88**	0.58	0.63	0.66	0.59	0.56	0.58
	k-fold validation	**0.8**	**0.85**	0.57	0.61	0.66	0.59	0.56	0.56
DNN	Train-test split	**0.83**	**0.88**	0.57	0.61	0.67	0.59	0.57	0.57
	k-fold validation	**0.82**	**0.89**	0.57	0.62	0.67	0.59	0.57	0.55

The values with accuracy are given in bold.

Table 7 shows the results of recall, precision, and F1-score for data balanced with ROS. Since performance of train-test split and k-cross validation classification are almost the same so we are reporting recall, precision, and F1-score of train-test split only. It can be observed that Binary Case 1 has best performance metrics for all cases followed by Flair (E-Sentiment). In Binary Case 1 also RF performed best, followed by DNN and DT, while SVM performed worst.

**Table 7:** Recall, Precision, F1-score for balanced (Random oversampling) data classification.

		Binary class 1		Binary class 2		Flair		3-class case	
		VADER	Textblob	VADER	Textblob	F-sentiment	E-sentiment	VADER	Textblob
Random forest	Recall	**0.95**	**0.99**	0.59	0.68	0.65	0.78	0.65	0.66
	Precision	**0.96**	**0.99**	0.59	0.67	0.64	0.76	0.63	0.63
	F1-score	**0.95**	**0.99**	0.59	0.67	0.63	0.76	0.63	0.64
SVM	Recall	0.77	**0.85**	0.56	0.6	0.58	0.65	0.52	0.52
	Precision	0.77	**0.85**	0.56	0.6	0.58	0.65	0.52	0.52
	F1-score	0.77	**0.85**	0.57	0.6	0.58	0.65	0.52	0.52
Decision tree	Recall	**0.8**	**0.86**	0.57	0.6	0.59	0.66	0.56	0.55
	Precision	**0.81**	**0.85**	0.57	0.62	0.59	0.66	0.56	0.56
	F1-score	**0.8**	**0.85**	0.57	0.61	0.59	0.66	0.56	0.56
DNN	Recall	**0.84**	**0.9**	0.58	0.62	0.6	0.67	0.57	0.56
	Precision	**0.84**	**0.89**	0.58	0.62	0.6	0.67	0.58	0.56
	F1-score	**0.84**	**0.89**	0.58	0.61	0.6	0.67	0.5	0.5

The values with accuracy are given in bold.

### Balanced data classification with SMOTE

Table 8 shows the classification accuracy results for fMRI data balanced with SMOTE. It can be observed that the general trend of results are same as for ROS results, with Binary Case 1 outperforming rest of the cases. However, the accuracy in general is lower than that of ROS for all cases. For example, for SMOTE balanced data accuracy with RF is 91%, while it was 99% for ROS. TextBlob performed better for all classifiers giving the best results for RF. In order to explore the influence of randomized labels, we classified the best case of fMRI data (Balanced data with SMOTE) using shuffled labels. The results are shown in Table 9.

**Table 8:** Accuracy for fMRI data classification with balanced (SMOTE) class labels.

		Binary class 1		Binary class 2		Flair		3-class case	
		VADER	Textblob	VADER	Textblob	F-sentiment	E-sentiment	VADER	Textblob
Random forest	Train-test split	**0.87**	**0.91**	0.58	0.64	0.72	0.61	0.58	0.57
	k-fold validation	**0.85**	**0.89**	0.57	0.63	0.7	0.6	0.55	0.55
SVM	Train-test split	**0.83**	**0.88**	0.58	0.61	0.69	0.59	0.55	0.55
	k-fold validation	**0.81**	**0.87**	0.57	0.6	0.67	0.57	0.52	0.53
Decision tree	Train-test split	0.68	0.71	0.54	0.57	0.6	0.54	0.45	0.56
	k-fold validation	0.67	0.7	0.52	0.54	0.58	0.54	0.43	0.44
DNN	Train-test split	**0.8**	**0.86**	0.57	0.61	0.66	0.57	0.52	0.52
	k-fold validation	**0.8**	**0.86**	0.56	0.61	0.65	0.58	0.52	0.52

The values with accuracy are given in bold.

Table 10 shows the recall, precision, and F1-scores for fMRI data classification balanced with SMOTE. Similar to ROS, Binary Case 1 results were best for all three performance metrics and for all classifiers. However, in this case DT provided worst scores while SVM provided worst scores for ROS.

**Table 9:** Accuracy for fMRI data (balanced with SMOTE) classification using shuffled labels.

	Binary class 1		Binary class 2		Flair		3-class case	
	VADER	Textblob	VADER	Textblob	F-sentiment	E-sentiment	VADER	Textblob
Random forest	0.51	0.50	0.50	0.49	0.50	0.51	0.33	0.32
SVM	0.51	0.50	0.49	0.50	0.49	0.50	0.33	0.33
Decision tree	0.50	0.50	0.49	0.49	0.50	0.50	0.34	0.33

**Table 10:** Recall, Precision, F1-score for balanced (SMOTE) data classification.

		Binary class 1		Binary class 2		Flair		3-class case	
		VADER	Textblob	VADER	Textblob	Fast sentiment	E sentiment	VADER	Textblob
Random forest	Recall	**0.87**	**0.91**	0.58	0.64	0.72	0.61	0.58	0.57
	Precision	**0.88**	**0.91**	0.58	0.64	0.73	0.61	0.59	0.58
	F1-score	**0.87**	**0.91**	0.58	0.64	0.71	0.61	0.58	0.57
SVM	Recall	**0.83**	**0.88**	0.58	0.61	0.69	0.59	0.55	0.55
	Precision	**0.83**	**0.88**	0.58	0.62	0.69	0.59	0.56	0.55
	F1-score	**0.83**	**0.88**	0.58	0.61	0.68	0.59	0.56	0.55
Decision tree	Recall	0.68	0.71	0.53	0.57	0.6	0.54	0.45	0.46
	Precision	0.68	0.72	0.53	0.57	0.6	0.54	0.45	0.45
	F1-score	0.68	0.71	0.53	0.57	0.6	0.54	0.45	0.45
DNN	Recall	**0.8**	**0.87**	0.57	0.61	0.66	0.57	0.51	0.52
	Precision	**0.81**	**0.87**	0.57	0.61	0.66	0.57	0.51	0.52
	F1-score	**0.8**	**0.87**	0.57	0.61	0.66	0.57	0.51	0.52

The values with accuracy are given in bold.

## Discussion

In this study, we performed sentiment classification using fMRI data obtained during movie watching. There are number of novel ideas/concepts that we explored for the first time in this study to obtain our results.

The first novelty of our study is generation of labels by performing sentiment analysis of the movie subtitles for fMRI data classification. These labels were generated using three sentiment analyzers; (1) VADER (2) TextBlob and (3) Flair. VADER and Textblob are lexicon-based analyzers, while Flair is AI-based. These analyzers perform best under different scenarios for sentiment analysis from text data. For example, VADER performs best on social media data, which is from Facebook, Twitter etc and TextBlob works best with formal language. Flair is very simple to use, it has pre-trained sentiment analysis models and it is trained on IMDB data. Our data (subtitles) was different from the types of data on which these analyzers work best due to which we did not have any performance benchmark available. In the absence of any such benchmark, we did similarity check of their results. We found that the labels generated were reasonably similar with most of them above 65%. Comparison results showed best similarity between VADER and Textblob, followed by similarity between E-Flair and F-Flair. It shows that the labels generation is largely dependent upon type of the sentiment analyzers. Being lexicon-based VADER and Textblob produced similar results, and being ML-based E-Flair and F-Flair produced similar results. Furthermore, we observed overall better similarity scores for Binary Case 1. These results indicate the importance of choice of right sentiment analyzer based on the type of data to be analyzed.

Next, we classified the subtitles using labels generated with sentiment analyzers to explore their success in classification of the data from which they were created. We use RF and SVM classifiers for this purpose and observed good classification accuracy for all cases with best ones for VADER and TextBlob generated labels compared to the labels generated by Flair (both E-Flair and F-Flair). This may indicate that pre-trained models of Flair are not suitable for sentiment analysis of movie subtitles. For VADER and TextBlob cases also, the classification accuracy was higher for the binary cases as compared to 3-Class classification. This maybe since the selected movie being a rom-com is not expected to have neutral contents (having neither positive nor negative contents), which was the class added for 3-Class classification (in addition to negative and positive classes). For binary case also, Binary Case 1 performed slightly better than Binary Case 2. Our results show that similar to Tweets and reviews, movie subtitles can also be successfully classified using sentiment analyzers.

After labels’ classification, we did classification of sentiments/emotions using imbalanced fMRI data using the labels generated with sentiment analysers. Similar to subtitles’ classification, Binary Case 1 outperformed Binary Case 2 and 3-Class Case for all classifiers. This similarity with the results of subtitles classification is promising since it validates our hypothesis that contents of the movie influence sentiments of the movie viewers similarly. Textblob performed best for binary cases, which may be since it works best for formal language usage. Our subtitles had narrations and dialogues, which can be counted as formal language. Consequently, labels generated with Textblob are aligned more with actual induced sentiments reflected in brain activation. VADER performed slightly worse than Textblob since it focuses on social media that contains emojis, repetitive words, and punctuation that are absent from subtitles. Binary Case 1 performed much better than Binary Case 2, which may indicate that the sentiments identified as neutral are more positive than negative in the selected movie. This makes sense since the movie we selected is a romantic comedy and is expected to have more positive nuances compared to negative ones.

Balancing the data improved overall classification accuracy for all cases, however, the overall accuracy pattern remained the same. Binary Case 1 performed best for balanced data (for both ROS and SMOTE) as well. After balancing, we classified the data with RF, SVM, DT, and DNN using simple train-test split and k-cross (k=10) validation. TextBlob binary labels performed best for RF giving an accuracy of 99%. In general also, for all classifiers and labels, TextBlob generated labels performed either as good as VADER or better than it, similar to imbalanced data classification. For ROS, TextBlob has an accuracy of 99% for RF and 88% for DNN model while for VADER we got 95% for RF and 83% for DNN. For SMOTE TextBlob had 91% accuracy for RF and for DNN model it had 86% accuracy. VADER also was able to achieve 87% for RF and 80% for DNN. It was also noted that the accuracies recorded for both train-test split and k-cross validation were very similar (within ± 0.01) showing that the method of data division did not influence the results.

The results reported with SMOTE data balancing had trend similar to the ROS having best results for Binary Case 1. In this case also the results for TextBlob labels and RF classifiers were best, however, the maximum accuracy obtained in this case was less (91%). This difference may be due to the reason that SMOTE is an improved form of random oversampling since it does not replicate but bring variety in the data samples. This would mean less overlap between train and test data compared to the ROS resulting in lower accuracy values.

In order to confirm that our results are not by chance, we randomized (shuffled) the labels and classified both subtitles and balanced fMRI data. The classification accuracies with randomized labels decreased a lot for both subtitles and fMRI showing the relevance of the subtitles’ generated labels to the sentiment classification from fMRI.

Overall for all cases, Binary Case 1 having neutral labels treated as positive outperformed all other cases for all classifiers. It may be since we used first 30 min of fMRI from a romantic comedy movie. The overall tone of the movie was positive during these 30 minutes and Binary Case 1 outperforming others reflects this fact. Furthermore, Textblob performed better than VADER and Flair since it is designed to work with formal language, which is used in movie subtitles. Successful classification of sentiments from fMRI data using subtitles-based labels show that the contents of naturalistic stimuli significantly contribute to the induced sentiments. Finding the contributions of various contents of movies such as video, audio, and dialogues can be beneficial at many levels such as identifying the differences of these contributions between healthy and disordered brain functions. Some recent studies have also identified this fact^13,77^, however, this field of study is still in its infancy and a lot can be explored.

The main limitation of our study was lack of related studies for comparison. Most of the studies in affective neuroscience domain use movie clips and not continuous movies as stimuli, which cannot be taken as naturalistic stimuli. Furthermore, automatic time-varying labeling of movies is not performed in any study. There are no universal indicators to validate our results, however, we observe certain pattern in our results (with highest classification accuracy for Binary Class 1) for all classifiers, which shows effectiveness and relevance of automatically generated labels. This is further validated by huge reduction in the accuracy for randomized/shuffled labels. Another limitation of our study was using just movie subtitles for labels generation instead of all contents such as video, audio, background music, and dialogue delivery. Additionally, we utilized only first 30 min of the movie, which does not reflect the dynamics of the whole movie.

Future studies can include labels based on combination of different contents of the movie and develop an adaptive model/algorithm that will incorporate the contributions of these components and separate out the stimuli-related and stimuli-independent (idiosyncratic) contributions in the emotions/sentiments. They can also use more than one movies from various genres for comparison purpose.

## Conclusion

Sentiment Analysis is widely used now a days for the analysis on customers reviews to check if the reviews are towards positive side or not by extracting sentiments from their comments/reviews. However, using the sentiment analysis to generate labels for brain data (during movie watching) is the innovative part of this project. We first generated labels through three sentiment analyzers (VADER, TextBlob, Flair). The sentiment analyzers used in our study work best in their own domains (none specifically for sentiment analysis of subtitles) and thus they gave the accuracy accordingly. However, there were reasonable similarities in the generated labels. We used these labels with the fMRI data to classify sentiments. The TextBlob generated labels gave best results since TextBlob is developed for formal language analysis, which is similar to subtitles. For classifier, the Random Forest performed best in all cases for binary classification. Randomized labels reduced the classification accuracies showing that the subtitles’ generated labels do indeed contain information pertinent to the sentiment related activations in the brain.

## Data Availability

The dataset analysed during the current study is open-access and available at the web link https://openneuro.org/datasets/ds002837/versions/2.0.0.
